# Risk of Crop Yield Reduction in China under 1.5 °C and 2 °C Global Warming from CMIP6 Models

**DOI:** 10.3390/foods12020413

**Published:** 2023-01-15

**Authors:** Feiyu Wang, Chesheng Zhan, Lei Zou

**Affiliations:** 1 Key Laboratory of Water Cycle and Related Land Surface Processes, Institute of Geographic Sciences and Natural Resources Research, Chinese Academy of Sciences, Beijing 100101, China; 2 Key Laboratory of Ecosystem Network Observation and Modeling, Institute of Geographic Sciences and Natural Resources Research, Chinese Academy of Sciences, Beijing 100101, China

**Keywords:** global warming, crop yield, risk, China

## Abstract

Warmer temperatures significantly influence crop yields, which are a critical determinant of food supply and human well-being. In this study, a probabilistic approach based on bivariate copula models was used to investigate the dependence (described by joint distribution) between crop yield and growing season temperature (T_GS_) in the major producing provinces of China for three staple crops (i.e., rice, wheat, and maize). Based on the outputs of 12 models from the Coupled Model Intercomparison Project Phase 6 (CMIP6) under Shared Socioeconomic Pathway 5–8.5, the probability of yield reduction under 1.5 °C and 2 °C global warming was estimated, which has great implications for agricultural risk management. Results showed that yield response to T_GS_ varied with crop and region, with the most vulnerable being rice in Sichuan, wheat in Sichuan and Gansu, and maize in Shandong, Liaoning, Jilin, Nei Mongol, Shanxi, and Hebei. Among the selected five copulas, Archimedean/elliptical copulas were more suitable to describe the joint distribution between T_GS_ and yield in most rice-/maize-producing provinces. The probability of yield reduction was greater in vulnerable provinces than in non-vulnerable provinces, with maize facing a higher risk of warming-driven yield loss than rice and wheat. Compared to the 1.5 °C global warming, an additional 0.5 °C warming would increase the yield loss risk in vulnerable provinces by 2–17%, 1–16%, and 3–17% for rice, wheat, and maize, respectively. The copula-based model proved to be an effective tool to provide probabilistic estimates of yield reduction due to warming and can be applied to other crops and regions. The results of this study demonstrated the importance of keeping global warming within 1.5 °C to mitigate the yield loss risk and optimize agricultural decision-making in vulnerable regions.

## 1. Introduction

The global surface temperature during the first two decades of the 21st century (2001–2020) has increased by 0.99 °C compared to the pre-industrial level (1850–1900), with a larger increase on land than in the ocean [1]. This warming trend is projected to continue in the following decades with rising greenhouse gas emissions, particularly in cultivated areas [2,3,4,5]. Given that global food demand is expected to double by the 2050s [3,6,7], global warming will pose more challenges to crop yield and food supplies for the next several decades. Therefore, it is crucial to estimate the possible changes in crop yield within the context of global warming.

Previous studies have shown that crop yields are affected by numerous climatic factors (e.g., temperature, precipitation, and drought) and their interactions [2,8,9,10,11,12,13]. Among these factors, temperature changes (i.e., warming trends) are expected to be more deterministic than others [14], and thus estimating temperature effects on crop yields is essential for climate change risk management. Generally, in most regions, higher temperatures can reduce crop yields by accelerating crop growth and shortening the growing period, or by exacerbating the negative impact of other factors on yield, such as warming-driven drought and compound dry-hot events [8,15,16,17,18]. However, warming may positively affect crop yields in other areas, such as those with heat deficits and those with adaptive measures (e.g., alteration of cultivar, planting dates, and irrigation types) [5,17,19,20,21]. Hence, the different mechanisms of temperature effects on crop growth lead to uncertainty in estimating possible yield changes under global warming.

Many approaches have been employed to study the climatic effect on crop yield, including field experiments, statistical regression, and crop model simulations [5,14,22,23,24,25]. For instance, Zhao et al. [14] investigated the temperature impact on yields of four crops (wheat, rice, maize, and soybean) based on four analytical methods and the authors found that temperature negatively affected yield at the global scale. Ray et al. [25] used a statistical crop time series for ~13,500 political units to analyze the variations in yields of maize, rice, wheat, and soybean caused by climate change and indicated that climate variability accounted for roughly one-third (~32–39%) of the observed yield variability. However, most previous studies have provided deterministic rather than probabilistic estimates of temperature effects on crop yield [26,27,28]. In practice, it is difficult to obtain an accurate estimate due to inevitable uncertainties in model structure or parameters, data quality, and incomplete consideration of the physical mechanisms related to crop growth [2,8,12,14,29]. In this case, the probability-based approach helps better characterize the yield-temperature relationship and its variation under warming conditions [30,31,32]. Among probabilistic models, copula-based models have been widely used in agriculture to explore the dependence between crop yields and climate variability (e.g., precipitation, soil moisture, solar radiation, and temperature) [11,23]. Based on the joint probability distribution of two individual variables (e.g., yield and temperature), the copula functions enable flexible estimation of the conditional probability of one variable when a certain threshold is exceeded for the other variable [33].

A new global temperature goal was recently established in the Paris Agreement to limit the increase in global temperature to 2 °C above pre-industrial levels, and preferably to 1.5 °C [34]. This goal aims to minimize the risk of climate change worldwide. Herein, we focused on the risk of yield reduction under 1.5 °C and 2 °C global warming, i.e., the probability of yield reduction in response to higher temperatures. Meanwhile, China is the second largest crop-producing country in the world, contributing to 17.4%, 21.9%, and 14.8% of the total global production of maize, rice, and wheat, respectively [12]. This indicates that crop yield in China is a matter of both domestic and global food supplies, especially given the threat of global warming and the continuously increasing population [27,35].

In this study, a copula-based approach is developed to model the joint probability distribution of crop yield and temperature for assessing the possible outcomes of yield changes under 1.5 °C and 2 °C global warming scenarios. This study was conducted on the main producing provinces corresponding to three staple crops (i.e., rice, wheat, and maize) in China, focusing on those provinces vulnerable to warming. The objectives of this study were to: (1) investigate the dependence between crop yield and temperature; (2) examine the yield sensitivity to different temperature conditions; and (3) estimate the risk of yield loss at 1.5 °C and 2 °C global warming targets.

## 2. Materials and Methods

### 2.1. Crop Yield and Meteorological Data

We obtained annual crop yield data (from 1995 to 2014) and crop production data (from 2015 to 2019) for rice, wheat, and maize for all provinces (or autonomous regions) from the China Agriculture Statistical Report, compiled by the Ministry of Agriculture and Rural Affairs of the People’s Republic of China. The top ten producing provinces for each crop were selected as the study area based on the average crop production in recent years (2015–2019). For each crop, the sum of production in the top ten producing provinces accounted for more than 80% of the total national production. The growing season and cropping system information for each crop-province pair were collected from previous studies and the agricultural atlas [36,37,38,39,40,41,42,43], as shown in Table 1. The long-term average annual yield and growing season temperature during the reference period are also shown in Table 1.

The observational temperature dataset during 1994–2014 was derived from a daily high-resolution (0.5° × 0.5°) meteorological dataset (i.e., CN05.1) [44] provided by the National Climate Center of the China Meteorological Administration. It should be noted that temperature data were collected from 1994 (one year ahead of crop yield data) because the growing season for winter wheat starts from the previous winter. This dataset was constructed based on interpolation from over 2416 station observations across China and has been widely used to evaluate climate model performance and analyze the climate characteristics of China [45,46,47]. It has proven to be a reliable reproduction of the historical climate in China [48,49]. The monthly temperatures were then calculated based on the daily data derived from the arithmetic mean. In addition, the simulated daily temperature data for the historical period and future scenario experiment were derived from 12 models of Coupled Model Inter-comparison Project Phase 6 (CMIP6), as listed in Table 2. This study focused on the high-emission shared socioeconomic pathway 5-8.5 (SSP5-8.5). All the model projections were bias-corrected and downscaled using the Bias Correction and Spatial Downscaling approach (BCSD), which has been widely used in the meteorological field [50,51,52].

**Figure 1:**
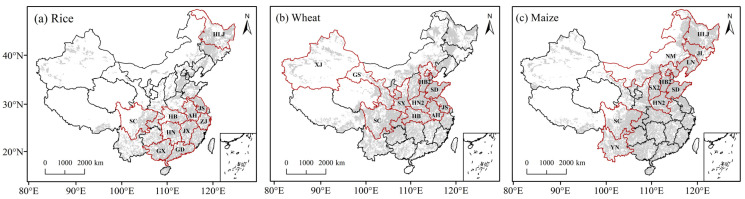
Main crop-producing provinces for (**a**) rice, (**b**) wheat, and (**c**) maize, respectively. The grey-shaded areas indicate the harvested areas. The provinces surrounded by red lines represent the top 10 producing provinces. The full names of the abbreviations for each province can be found in Table 1.

The spatially weighted average temperature for each province was calculated based on the weight defined by the crop harvested area mapping in 2000, acquired from the data center of the global spatial production allocation model (SPAM) (http://mapspam.info/data/, accessed on 11 August 2022) (Figure 1). According to the growing season information (Table 1), the annual growing season temperature (T_GS_) was calculated from the average monthly temperatures during the growing season. The first-difference method was used to detrend the yield and T_GS_ data to eliminate the confounding influence of long-term variations, such as changes in crop management and technological advancement [9,53,54].

### 2.2. Copula-Based Model

Copula functions are powerful tools to describe the dependence structure between random variables [33,55]. In this study, we used the bivariate copula model to construct the joint probability distribution of temperature (*X*) and crop yield (*Y*) based on their univariate distributions. According to Sklar’s theorem [56], a joint cumulative distribution function (CDF) can be expressed as follows:

(1) $$F_{X,Y}(x,y)=C[u,v]$$

where *u* and *v* denote the marginal distribution functions of *X* and *Y*, which are uniformly distributed in the domain of 0 to 1 [33], and copula *C* describes the bivariate joint CDF of *u* and *v*.

The most commonly used copula families in meteorological and hydrological studies are elliptical and Archimedean copulas [11,23,57]. Herein, two popular elliptical copulas (i.e., Gaussian and t) and three Archimedean copulas (i.e., Frank, Clayton, and Gumbel) were chosen to model the joint probability distribution between temperature (*X*) and yield (*Y*), as listed in Table 3. Different copulas reflect different characteristics of the overall dependence structure and the tail dependence, with the latter describes the joint distribution between the extreme values of the variables, which is important for risk analysis. Among these five copulas, the Gaussian, t, and Frank copulas describe symmetric dependence structures, i.e., the same degree of dependence in the upper and lower tails (which correspond to extreme values), but with different behaviors at the corners of quadrants. In contrast, the Clayton and Gumbel copulas characterize an asymmetric tail dependence with a greater dependence in the lower and upper tails, respectively.

Based on the joint distribution between *X* and *Y*, the conditional probability of *Y* dropping below a certain threshold (*Y* < *y*) under different *X* conditions (*X* = *x*_1_, *x*_2_, …), i.e., P(*Y* < *y* | *X* = *x*), can be estimated. The conditional probability density function (PDF) can be expressed as follows [32,58]:

(2) $$f_{Y|X}(y|x)=c\left[u,v\right]\cdot f_{Y}(y)$$

where *c* denotes the joint PDF of the copula function, and $f_{Y}\left(y\right)$ denotes the PDF of the marginal distribution for *Y*. Once the conditional PDF is determined, the probability P(*Y* < *y* | *X* = *x*) can be calculated as the area under the PDF curve within the interval (-∞, *y*]. Obviously, the area under the whole PDF curve is always exactly 1.

The data processing flow is as follows. First, the marginal distributions were fitted to the detrended yield (∆Yield) and T_GS_ (∆T_GS_). Second, the five bivariate copulas were fitted to ∆Yield and ∆T_GS_ data, and the optimal copula was selected based on the comprehensive goodness of fit measures, including the Akaike information criterion (AIC), Bayesian information criterion (BIC), root mean square error (RMSE), and Nash-Sutcliffe efficiency (NSE) [59]. The conditional PDFs for different ∆T_GS_ conditions were then determined. Finally, the probability of yield reduction (i.e., ∆Yield < 0) for each warming condition (i.e., 1.5 °C and 2 °C global warming) was estimated. All data processing and analysis work was implemented based on the MATLAB platform.

### 2.3. Dependence Measure

Spearman’s rank correlation coefficient (rho) is a measure that assesses the extent to which a monotonic function can describe the dependence between two random variables, *X* and *Y*. Since it is defined by the rank of given data rather than the data itself, it remains scale-invariant under strictly increasing transformations of the random variables [33]. Hence, when working with copulas, Spearman’s rho is more appropriate than Pearson’s correlation coefficient (which measures the linear dependence between random variables). The Spearman’s rho (*ρ_S_*) for random variables *X* and *Y* can be expressed by the copula *C*(*u*,*v*) as follows [33]:

(3) $$\rho_{S}=12\iint_{{\left[0,1\right]}^{2}}uvdC(u,v)-3$$

where *u* and *v* denote the marginal distribution functions of *X* and *Y*, as mentioned earlier.

### 2.4. Timing of Reaching the Global Warming Targets

**Figure 2:**
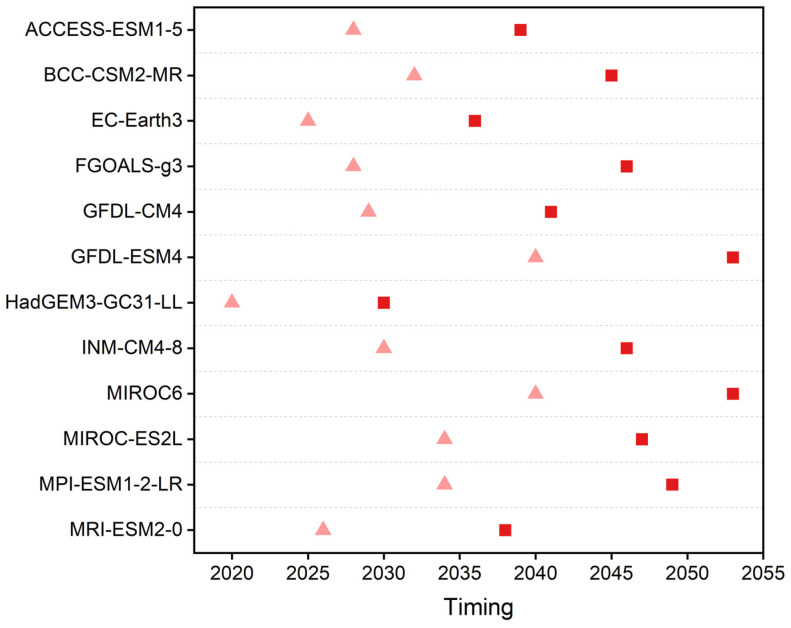
Timing to reach 1.5 °C (pink triangles) and 2 °C (red squares) global warming targets under SSP5-8.5. The year denotes the central timing of a 20-year time window.

The global warming targets of 1.5 °C and 2 °C refer to global mean surface temperature (GMST) increases of 1.5 °C and 2 °C above the pre-industrial level. Since the reference period was defined as 1995–2014 in this study, a 20-year time window was used to determine the timing of reaching the 1.5 °C and 2 °C global warming targets. The specific timing of reaching the global warming targets was then determined as the first time window when the GMST of each climate model reached 1.5 °C and 2 °C above the pre-industrial equivalent. As shown in Figure 2, this timing under SSP5-8.5 varied with the climate model. Thus, we used a multi-model ensemble mean to reduce the uncertainty caused by differences among models to analyze the yield response to future warming.

## 3. Results

### 3.1. Dependence between Yield and Growing Season Temperature

**Figure 3:**
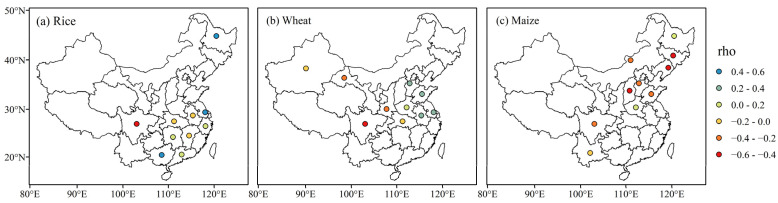
Spearman’s rank correlation coefficient (rho) between the detrended yield and temperature.

Figure 3 shows the Spearman’s rho between the detrended T_GS_ and yield for each crop and province. For rice, temperature and yield were significantly negatively correlated in Sichuan province (rho = −0.52, *p* < 0.05), while they were significantly positively correlated in Heilongjiang (rho = 0.51, *p* < 0.05) and Jiangsu (rho = 0.45, *p* < 0.05) provinces as well as Guangxi Zhuang Autonomous Region (rho = 0.42, *p* < 0.05). For wheat, temperature and yield were negatively correlated in the northwestern and southwestern provinces, with the lowest correlation coefficient of −0.55 (*p* < 0.05) in Sichuan province, while positive correlations were observed in northern China and the Yangtze River Delta provinces. In contrast, for maize, temperature and yield were negatively correlated for all provinces except Heilongjiang and Henan, with the lowest correlation coefficient of −0.48 (*p* < 0.05) in Liaoning province. The correlations between T_GS_ and yield are consistent with those in previous studies [27,40,42,43,60,61,62,63]. These results indicated that the dependence between yield and T_GS_ varied with crop and region. Overall, a negative correlation between yield and T_GS_ was observed in about half of the rice- and wheat-producing provinces and in the vast majority of the maize-producing provinces.

Five copulas were fitted to the detrended T_GS_ and yield data, and then the optimal copula for each crop and province was selected according to AIC, as shown in Table 4. It can be seen that Archimedean copulas were more suitable for describing the joint distributions between T_GS_ and yield in most rice-producing provinces. For wheat-producing provinces, the optimal copulas were equally divided between elliptical and Archimedean copulas, while for maize-producing provinces, elliptical copulas dominated. Overall, there was a tail dependence between ∆T_GS_ and ∆Yield for more than 1/3 of the major producing provinces, indicating a higher probability of the simultaneous occurrence of extremes in temperature and yield.

**Figure 4:**
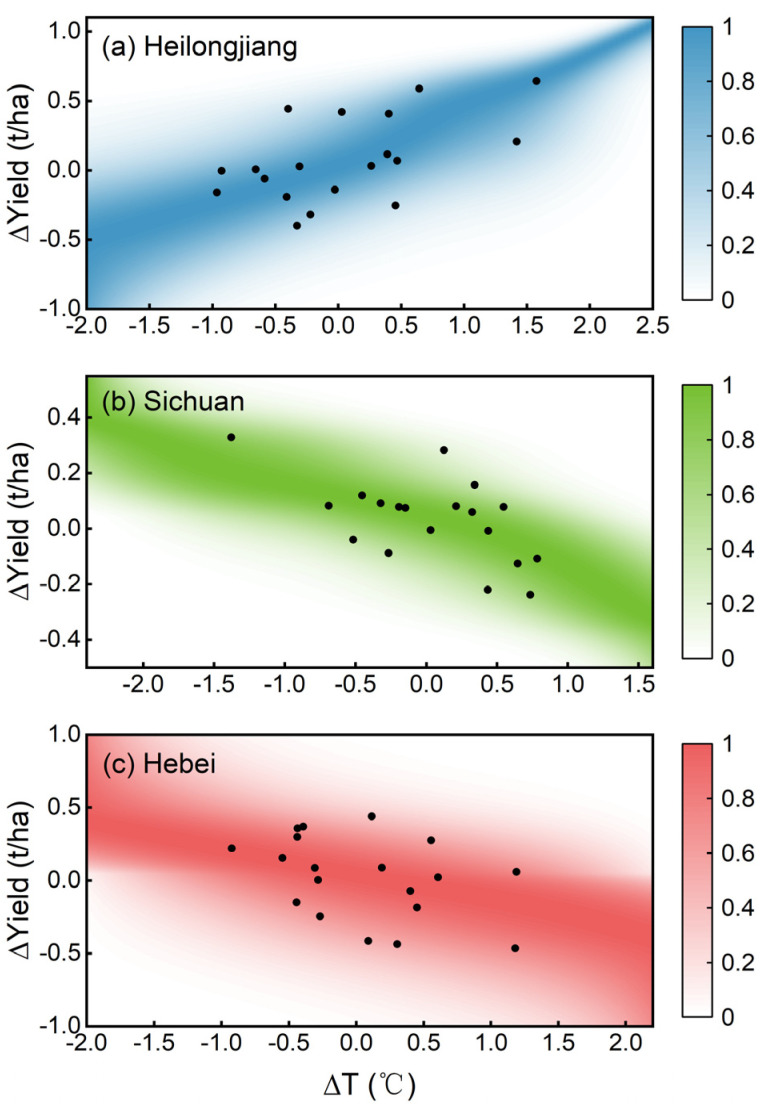
Joint distribution (normalized between 0 to 1) between detrended temperature (∆T_GS_) and yield (∆Yield) during the reference period for (**a**) Heilongjiang province (rice), (**b**) Sichuan province (wheat), and (**c**) Hebei province (maize), respectively. The colored pixels on the z-axis in each panel represent the probability density function (PDF) at a given ∆T_GS_-∆Yield pair, with 1 denoting the highest density and 0 denoting the lowest density. The black dots show the location of the observed ∆Yield at different ∆T_GS_.

For visualization, a typical province was chosen for each crop to illustrate the dependence characteristics between ∆T_GS_ and ∆Yield. They are Heilongjiang province (rice), Sichuan province (wheat), and Hebei province (maize), which belong to the cold-temperate and temperate continental monsoon climate, subtropical monsoon climate, and temperate continental monsoon climate zones, respectively, indicating that they are under different heat conditions. Figure 4 compares the estimated ∆Yield distribution with the observed ∆Yield under different ∆T_GS_ during the reference period. Specifically, Heilongjiang province showed an upper tail dependence based on Gumbel copula, reflecting a greater probability of higher ∆Yield with higher ∆T_GS_ (Figure 4a). Taking wheat in Sichuan and maize in Hebei as examples, the joint distribution of ∆T_GS_ and ∆Yield based on Gaussian copula exhibited a symmetric dependence structure but with tail independence (Figure 4b,c). As seen, most of the ∆Yield fell in the high-density area of the PDF in all panels, indicating that the estimated distributions were reliable for describing the dependence between temperature and yield.

### 3.2. Conditional Probability of Yield Reduction under Different Warming Conditions

**Figure 5:**
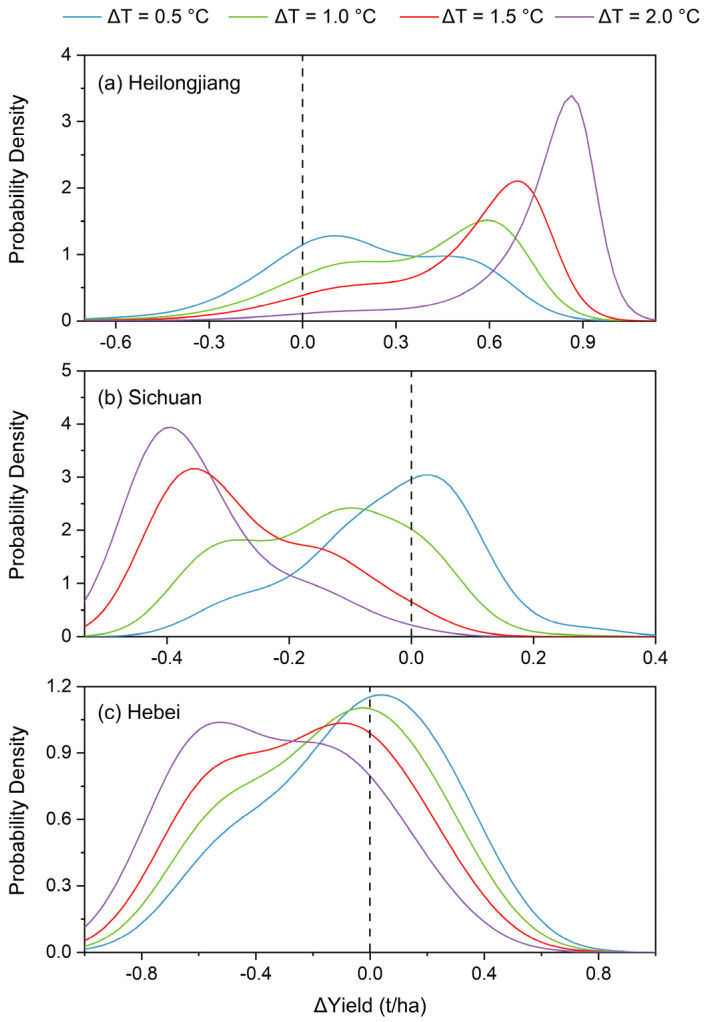
Conditional probability distribution of yield variation (∆Yield) given four warming conditions (∆T_GS_ = 0.5, 1, 1.5, and 2 °C) for (**a**) Heilongjiang province (rice), (**b**) Sichuan province (wheat), and (**c**) Hebei province (maize), respectively.

Based on the joint distribution of ∆T_GS_ and ∆Yield, the conditional probabilities of yield variation under different warming conditions (herein ∆T_GS_ = 0.5 °C, 1 °C, 1.5 °C, and 2 °C) were estimated to reveal the sensitivity of yield to warming. As shown in Figure 5a, the conditional probability distribution of ∆Yield for rice in Heilongjiang province became more left-skewed with enhanced warming, indicating that yield was more likely to increase, or in other words, less likely to decrease, with warmer temperatures. By contrast, the conditional probability distribution of ∆Yield for wheat in Sichuan province and maize in Hebei province became more right-skewed as warming intensified (Figure 5b,c), suggesting a greater likelihood of yield reduction with warmer temperatures. These results indicated that the probability distribution of yield had different skewness and kurtosis under different warming conditions and varied with crop and province.

Based on the conditional PDF of yield for each crop and province, we estimated the conditional probability of yield reduction (i.e., ∆Yield < 0) under different warming conditions by calculating the area under the PDF curve within the interval (-∞, 0]. According to the correlation between yield and temperature (Figure 3), we divided the producing provinces of each crop into two parts, one with a negative temperature-yield correlation and the other with a positive temperature-yield correlation. The negative/positive temperature-yield correlation reflected the potential benefit/threat of warming on yield. Hence, provinces with a negative/positive correlation between temperature and yield were referred to as vulnerable/non-vulnerable provinces thereafter.

**Figure 6:**
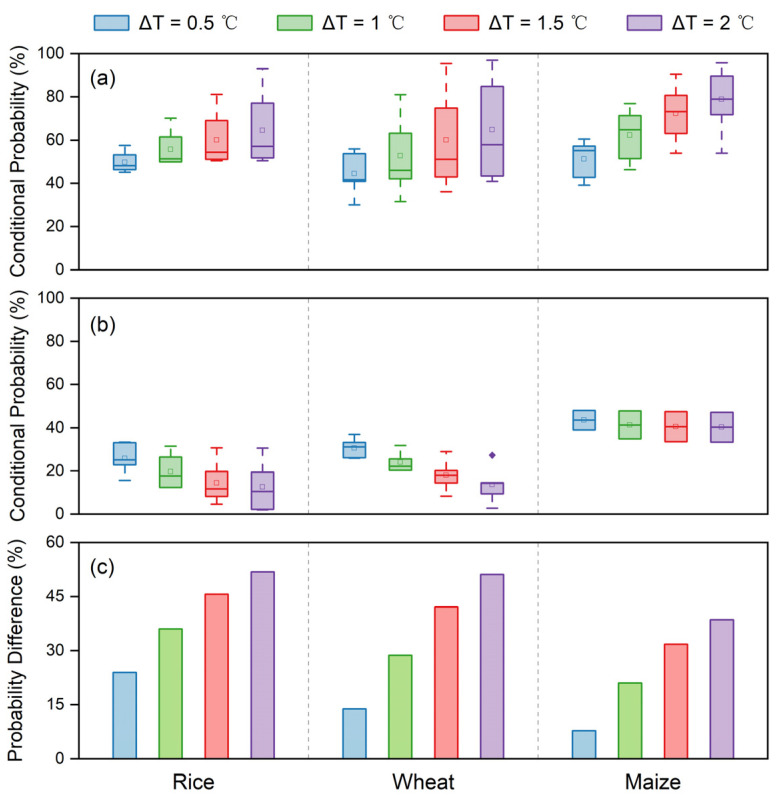
Conditional probability distribution of yield variation (∆Yield) given four warming conditions (∆T_GS_ = 0.5, 1, 1.5, and 2 °C) for provinces where yield and temperature were (**a**) negatively correlated and (**b**) positively correlated, respectively. The mean difference of conditional probability between (**a**,**b**) is shown in (**c**).

Figure 6 shows the overall probability of yield reduction for each crop under the four warming conditions. Overall, the yield reduction probability under warming for all three crops was higher in the vulnerable provinces than in the non-vulnerable provinces, with maize having a greater probability of yield reduction than rice and maize. It should be noted that the difference in the probability of yield reduction for maize was smaller between vulnerable and non-vulnerable provinces than for rice and wheat (Figure 6c). This suggested that maize was at greater risk of warming-driven yield reduction than rice and wheat. Specifically, the upward gradient in the probability of maize yield reduction was greater in the vulnerable provinces than the downward gradient in the non-vulnerable provinces, indicating that the sensitivity of maize yield to warming was higher in the vulnerable provinces.

### 3.3. Future Global Warming and its Effect on Yield Reduction

**Figure 7:**
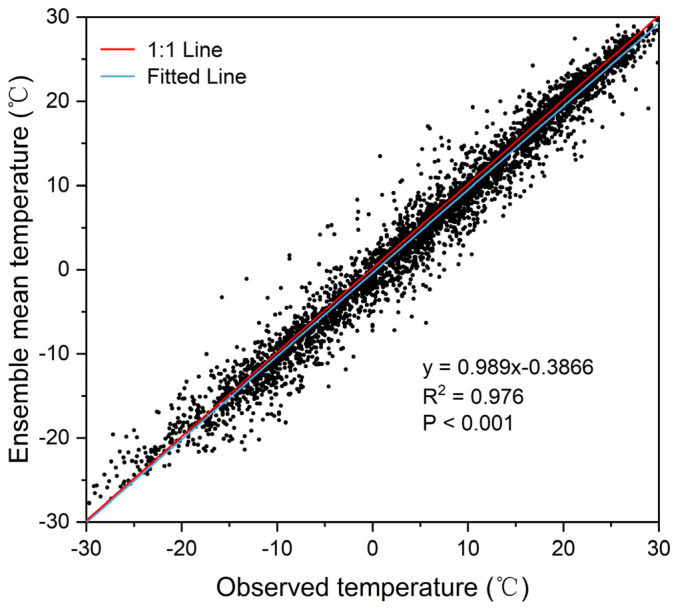
Validation of the ensemble mean temperature of the 12 CMIP6 models against the observed temperature. Each black dot represents a randomly selected grid point within China.

**Figure 8:**
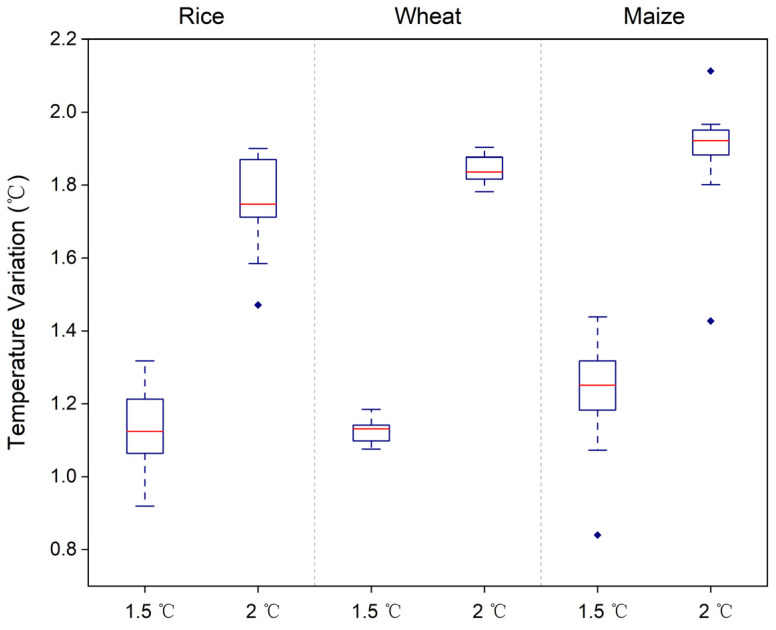
Growing season temperature variations in main crop-producing provinces at 1.5 °C and 2 °C global warming under SSP5-8.5 compared to the reference period (1995–2014).

The bias-corrected and downscaled temperature data were validated in the reference period by randomly selecting grid points within China. As shown in Figure 7, the multi-model ensemble mean of temperature presented a good agreement with the observed temperature (R^2^ = 0.976, *p* < 0.001), indicating that the corrected simulation data could reproduce the temperature variation and therefore were suitable for future warming prediction. The future T_GS_ variations for each crop and province were then calculated at 1.5 °C and 2 °C global warming under SSP5-8.5 compared to the reference period. Overall, the magnitude of variation in T_GS_ varies with crop and is ranked under both global warming conditions: maize > wheat > rice (Figure 8). Notably, the difference in T_GS_ variation between the 1.5 °C and 2 °C global warming will exceed 0.5 °C, indicating that the increasing gradient in T_GS_ between the two warming conditions is greater than that of GMST.

**Figure 9:**
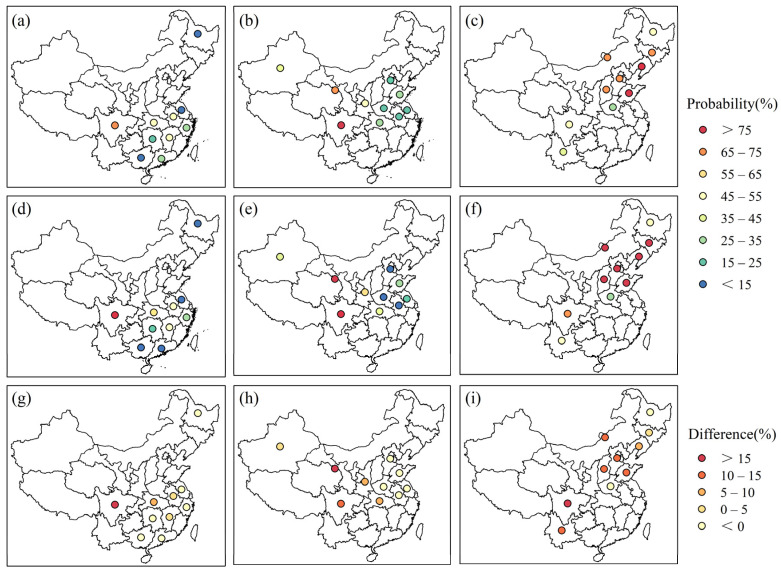
Probability of yield reduction for rice (**first** column, **a**,**d**,**g**), wheat (**second** column, **b**,**e**,**h**), and maize (**third** column, **c**,**f**,**i**) at 1.5 °C (**top** row, **a**–**c**) and 2 °C (**middle** row, **d**–**f**) global warming under SSP5-8.5. The difference in the probability of yield reduction between 1.5 °C and 2 °C global warming is shown in the bottom row.

Figure 9 shows the conditional probability of yield reduction (∆Yield < 0) for each crop and province estimated from the ensemble mean T_GS_ variation (∆T_GS_) at 1.5 °C and 2 °C global warming under SSP5-8.5. At the 1.5 °C global warming, the yield reduction probability will be 11–71%, 18–84%, and 34–87% among the main producing provinces for rice, wheat, and maize, respectively (Figure 9a–c). Overall, the spatial pattern of the yield reduction probability is consistent for both warming conditions. The most vulnerable crop-provinces cases under warming are rice in Sichuan province, wheat in the Sichuan and Gansu provinces, and maize in Shandong, Liaoning, Jilin, Nei Mongol, Shanxi, and Hebei provinces, in line with the spatial pattern of the temperature-yield correlation (Figure 3). These provinces should be prioritized for developing climate adaptation strategies.

When additional global warming of 0.5 °C occurs (i.e., the 2 °C global warming condition), the probability of yield reduction will increase by 2–17%, 1–16%, and 3–17% for rice, wheat, and maize, respectively, in the vulnerable provinces, while declining to different degrees in the non-vulnerable provinces (Figure 9g–i). This suggests that the additional warming would pose a greater risk of yield reduction in the vulnerable provinces, while mitigating the yield reduction risk in non-vulnerable provinces. For instance, the risk of rice yield reduction will increase by 17% in Sichuan province, while it will decrease by 8% in Heilongjiang province.

## 4. Discussion

### 4.1. Crop Yield Response to Warming Conditions

The dependence characteristics between the detrended T_GS_ (∆T_GS_) and yield (∆Yield) showed an apparent spatial heterogeneity (Figure 3 and Table 4), indicating that yield response to temperature varied with crop and region. For example, rice yield and temperature were negatively correlated in Sichuan province, while they were positively correlated in Heilongjiang and Jiangsu provinces; the temperature was positively correlated with wheat yield and negatively correlated with maize yield in Shandong province. These results were broadly consistent with previous studies conducted at different scales over China, though to different degrees [21,39,40,64,65,66]. Generally, the warming effect on yield is twofold and closely related to the optimum temperature for crop growth [67]. On the one hand, warming can inhibit crop growth, especially during the heading-flowering stage, which shortens the growth period and thus reduces biomass accumulation [53,66,68,69]. For example, Sichuan province is relatively abundant in heat, so yields for all three crops were under warming stress (Figure 3). On the other hand, for some heat-deficient regions (e.g., Heilongjiang province), warming can converge the temperature to the optimum temperature for crop growth, thus enhancing photosynthesis and increasing biomass [21,64,66,70]. In addition, yield response to temperature is also influenced by other external factors, such as water supply [71]. For example, a study on U.S. maize showed that precipitation substantially altered the magnitude of temperature-driven yield changes [8]. A global-scale study indicated that the compound dry-hot condition had a greater impact on maize yields than the individual hot condition [72].

Based on the dependence between yield and temperature, we divided all provinces into two parts, those threatened by warming (vulnerable provinces) and those benefiting from it (non-vulnerable provinces). This study focused on the risk of yield reduction in vulnerable provinces. Figure 9g–i shows that an additional 0.5 °C of global warming will increase the yield reduction risk in vulnerable provinces by 2–17%, 1–16%, and 3–17% for rice, wheat, and maize, respectively. Since provinces vulnerable to warming account for about half of the major rice- and wheat-producing provinces and most maize-producing provinces (Figure 3), the risk of yield reduction in these provinces would threaten China’s agricultural productivity and total crop production. Hence, it is necessary to limit global warming to 1.5 °C to avoid the adverse effects of global warming on crop yields and thus protect food supplies.

Overall, the copula-based models proved to be an effective tool for investigating the temperature-yield relationship in different cropping systems and regions of China. Unlike the deterministic estimates of previous studies, the copula model can provide yield distribution given any temperature condition and further offer probabilistic estimates of yield loss risk. This can help farmers and stakeholders manage agricultural operations to meet the complex challenges of future climate change [73]. Previous studies have shown that copula can be flexibly applied to explore the relationship between yield and other yield-related climatic factors (e.g., precipitation and drought) [11,23,73]. Furthermore, by extending the bivariate model to a trivariate model, it is possible to estimate the yield loss risk under a combination of two climatic conditions (e.g., temperature and precipitation/drought) [72,74].

### 4.2. Uncertainties and Limitations

There are some uncertainties and limitations in this study. First, the dependence between temperature and yield was related to the chosen reference period and the growing period of a given crop, which may influence the trend and magnitude of yield changes under different temperature conditions. Second, this study only considered the effect of temperature on yield, but the yield is influenced by the combined effects of multiple climatic factors, such as drought, CO_2_ concentration, and extreme events [5,13,27,66]. For instance, with and without considering the effect of CO_2_ fertilization, yield reduction was projected to be less than 15% and 14% for maize and wheat in most areas of China under the 2 °C global warming condition [66]. Third, this study assumed that the temperature-yield relationship obtained in the reference period remained unchanged in the future. However, adaptive measures and technological improvements (e.g., changes in planting time and cultivars, irrigation, and fertilization) can partly offset the adverse effect of climate change on yield and thus alter the temperature-yield relationship [24,65,75,76]. Finally, uncertainties may derive from the model structure and parameters, the spatial and temporal scales, and other factors [27,29,76]. As a result, the probability of yield reduction under global warming may be underestimated or overestimated. However, despite these uncertainties, this study provided a reasonable estimate of the yield loss risk in China’s major crops and producing provinces under global warming. Future work can be extended to the county scale and incorporate other influencing factors into the copula model to obtain more accurate estimates of yield loss risk under global change.

## 5. Conclusions

This study used a probabilistic approach based on bivariate copulas to explore the dependence between yield and growing season temperature in major producing provinces of China for three staple crops, i.e., rice, wheat, and maize. The probability of yield reduction for each crop and province under 1.5 °C and 2 °C global warming conditions was then estimated based on the joint distribution of yield and temperature obtained from the optimal copulas. The main conclusions are as follows:

(1) The dependence between yield and growing season temperature varied with crop and region. Overall, Archimedean/elliptical copulas provided the best fits of joint distribution between T_GS_ and yield in most rice-/maize-producing provinces. There were four rice-producing provinces, five wheat-producing provinces, and eight maize-producing provinces vulnerable to warming pressures. The most vulnerable crop-province cases were rice in Sichuan province, wheat in the Sichuan and Gansu provinces, and maize in the Shandong, Liaoning, Jilin, Nei Mongol, Shanxi, and Hebei provinces.
(2) The yield reduction probability under warming was overall higher in vulnerable provinces than in non-vulnerable provinces, with maize having a greater yield reduction risk than rice and wheat. The sensitivity of maize yield to warming gradient was higher in vulnerable provinces than in non-vulnerable provinces.
(3) From 1.5 °C to 2 °C global warming, an additional 0.5 °C of warming could increase the risk of yield reduction in vulnerable provinces by 2–17%, 1–16%, and 3–17% for rice, wheat, and maize, respectively.

## Figures and Tables

**Table 1:** Crop-producing information for each crop-province pair.

Type	Province	Short Name	Growing Season	Cropping System
Rice	Heilongjiang	HLJ	May-September	Single rice
	Jiangsu	JS	April-October	Single rice
	Zhejiang	ZJ	April-November	Double rice
	Anhui	AH	April-November	Double rice
	Jiangxi	JX	April-November	Double rice
	Hubei	HB	April-November	Double rice
	Hunan	HN	April-November	Double rice
	Guangdong	GD	March-November	Double rice
	Guangxi	GX	March-November	Double rice
	Sichuan	SC	April-October	Single rice
Wheat	Hebei	HB2	October-May	Winter wheat
	Jiangsu	JS	November-May	Winter wheat
	Anhui	AH	November-April	Winter wheat
	Shandong	SD	September-June	Winter wheat
	Henan	HN2	September-June	Winter wheat
	Hubei	HB	November-April	Winter wheat
	Sichuan	SC	November-May	Winter wheat
	Shaanxi	SX	October-June	Winter wheat
	Gansu	GS	October-June	Winter wheat
	Xinjiang	XJ	October-June	Winter wheat
Maize	Hebei	HB2	June-September	Summer maize
	Shanxi	SX2	May-September	Spring maize
	Nei Mongol	NM	May-September	Spring maize
	Liaoning	LN	May-September	Spring maize
	Jilin	JL	May-September	Spring maize
	Heilongjiang	HLJ	May-September	Spring maize
	Shandong	SD	June-September	Spring maize
	Henan	HN2	June-September	Spring maize
	Sichuan	SC	June-September	Summer maize
	Yunnan	YN	June-September	Summer maize

**Table 2:** Information of 12 CMIP6 models used in this study.

Model	Country	Modeling Center	Resolution (lat × lon)
ACCESS-ESM1-5	Australia	Commonwealth Scientific and Industrial Research Organization and Bureau of Meteorology	1.25° × 1.875°
BCC-CSM2-MR	China	Beijing Climate Center	1.125° × 1.125°
EC-Earth3	Europe	EC-EARTH consortium	0.7° × 0.7°
FGOALS-g3	China	State Key Laboratory of Numerical Modeling for Atmospheric Sciences and Geophysical Fluid Dynamics (LASG), Institute of Atmospheric Physics, Chinese Academy of Sciences	2.25° × 2°
GFDL-CM4	USA	NOAA Geophysical Fluid Dynamics Laboratory	1.0° × 1.25°
GFDL-ESM4			
HadGEM3-GC31-LL	UK	Met Office Hadley Centre	1.25° × 1.875°
INM-CM4-8	Russia	Institute for Numerical Mathematics, Russian Academy of Science	1.5° × 2°
MIROC6	Japan	National Institute for Environmental Studies, University of Tokyo	1.4° × 1.4°
MIROC-ES2L			2.8° × 2.8°
MPI-ESM1-2-LR	Germany	Max Planck Institute for Meteorology	1.875° × 1.875°
MRI-ESM2-0	Japan	Meteorological Research Institute	1.125° × 1.125°

**Table 3:** Summary of five commonly used bivariate copulas.

Name	Function	Parameter Range
Gaussian	$C(u,v)=\int_{-\infty }^{\Phi^{-1}(u)}\int_{-\infty }^{\Phi^{-1}(v)}\frac{1}{2\pi \sqrt{\left(1-\theta^{2}\right)}}\exp\left\{-\frac{x^{2}+y^{2}-2\theta xy}{2(1-\theta^{2})}\right\}dxdy$	θ∈(−1,1)
t	$C(u,v)=\int_{-\infty }^{t_{v}{}^{-1}(u)}\int_{-\infty }^{t_{v}{}^{-1}(v)}\frac{1}{2\pi \sqrt{1-\theta^{2}}}\exp{\left\{1+\frac{x^{2}+y^{2}-2\theta xy}{v(1-\theta^{2})}\right\}}^{-\frac{v+2}{2}}dxdy$	θ∈(−1,1)
Clayton	$C(u,v)={\left(u^{-\theta }+v^{-\theta }-1\right)}^{-1/\theta }$	θ∈[0,∞)
Frank	$C(u,v)=-\frac{1}{\theta }\ln\left[1+\frac{\left(e^{-\theta u}-1\right)\left(e^{-\theta v}-1\right)}{e^{-\theta }-1}\right]$	θ∈R\0
Gumbel	$C(u,v)=\exp\left\{-{\left[{\left(-\ln u\right)}^{\theta }+{\left(-\ln v\right)}^{\theta }\right]}^{\frac{1}{\theta }}\right\}$	θ∈[1,+∞)

**Table 4:** Optimal copula for each crop and province during the reference period (1995–2014).

Rice			Wheat			Maize		
Province	Copula	AIC	Province	Copula	AIC	Province	Copula	AIC
HLJ	Gumbel	−129.48	HB2	Gaussian	−138.18	HB2	Gaussian	−124.70
JS	Clayton	−140.28	JS	Gumbel	−129.99	SX2	Gaussian	−123.97
ZJ	Clayton	−125.73	AH	Gumbel	−129.60	NM	Gaussian	−139.33
AH	Frank	−122.31	SD	Frank	−133.21	LN	Gaussian	−128.61
JX	Frank	−132.39	HN2	Gumbel	−140.42	JL	t	−127.13
HB	Gaussian	−138.04	HB	t	−129.41	HLJ	Gaussian	−130.76
HN	Clayton	−127.42	SC	Gaussian	−126.49	SD	Gaussian	−130.69
GD	Gumbel	−130.47	SX	Gaussian	−137.39	HN2	Clayton	−141.53
GX	Gaussian	−138.24	GS	Gaussian	−127.69	SC	Gaussian	−129.09
SC	Gaussian	−129.33	XJ	Frank	−134.41	YN	Gaussian	−140.45

## Data Availability

The datasets used or analyzed during the current study are available on reasonable request.
